# Application of the chocolate balloon (restrictive dilatation technique) in vascular preparation for arterial angioplasty of the lower limbs

**DOI:** 10.3389/fsurg.2025.1528231

**Published:** 2025-03-07

**Authors:** Zhong Hongzhao, Zhang Dawei, Zhao Bo, Yang Xuesong, Li Huihan, Hao Qingzhi, Song Longyu

**Affiliations:** ^1^ The First Clinical College of Shandong University of Traditional Chinese Medicine, Jinan, Shandong, China; ^2^ Department of Vascular Surgery, Affiliated Hospital of Shandong University of Traditional Chinese Medicine, Jinan, Shandong, China

**Keywords:** chocolate balloon, lower limb artery disease, endovascular treatment, retrospective study, restricted expansion

## Abstract

**Objective:**

This study aims to compare the immediate clinical effects of the chocolate balloon and the conventional balloon in endovascular angioplasty of the lower limbs.

**Methods:**

Clinical data were retrospectively collected from a single center, including 117 patients with lower limb arterial lesions treated from January to December 2021 and 112 patients treated from January to December 2023 at our center. The comparison focused on the incidence of vascular dissection after balloon dilatation, with secondary endpoints including the stent implantation rate.

**Results:**

In both groups, the success rate was 100%. Dissection formation rates in the chocolate balloon and conventional balloon groups were 20.5% vs. 17.5%, respectively. Non-flow-limiting dissection formation rates were 14.7% vs. 4.8% (*P* < 0.05), while severe dissection rates were 5.8% vs. 12.7% (*P* < 0.05). Stent implantation rates were 9.0% in the chocolate balloon group and 18.3% in the conventional balloon group (*P* < 0.05).

**Conclusion:**

The use of chocolate balloons resulted in a lower incidence of severe dissection and reduced the stent implantation rate compared to conventional balloons. It effectively prepares complex multiple lesions of lower limb arteries in real-world scenarios.

## 1 Introduction

Lower extremity artery disease encompasses a variety of conditions resulting from stenosis and occlusion of the lower extremity arteries, including thrombosis, arteriosclerosis, occlusion, vasculitis, and others. In its early stages, patients may experience intermittent claudication, progressing to resting pain and potentially leading to ulcers and gangrene as the disease advances. Currently, lower limb artery disease is characterized by high incidence, disability rates, and mortality (1), with its prevalence increasing with age (2) Percutaneous transluminal angioplasty (PTA) remains the primary clinical approach for treating lower limb artery diseases (3), often combined with the use of nickel-titanium stents to achieve immediate luminal patency. However, long-term patency rates are still unsatisfactory due to factors such as endovascular hyperplasia (4). So the utilization of drug-coated balloons (DCB) has shown promise in improving the long-term patency rates of lower limb arterial lumen therapy (5–7). Severe calcification and long chronic occlusive (CTO) lesions pose challenges, as predilation before DCB release during PTA treatment may increase the risk of significant vascular dissection. Consequently, many cases of severe dissections necessitate subsequent stent implantation, resulting in a notable rate of remedial stent deployment. The chocolate balloon, engineered with restrictive dilation technology, aims to reduce overall vascular trauma. A study demonstrated its immediate efficacy in minimizing fluid dissection formation, consequently reducing the need for remedial stents and lowering the target lesion vascular remodeling rate (8). However, there is limited research comparing the efficacy of the chocolate balloon vs. a conventional balloon in vascular preparation during PTA. Therefore, this study seeks to compare the clinical outcomes of lower limb artery disease treatment using a conventional balloon at our center.

**Figure 1:**
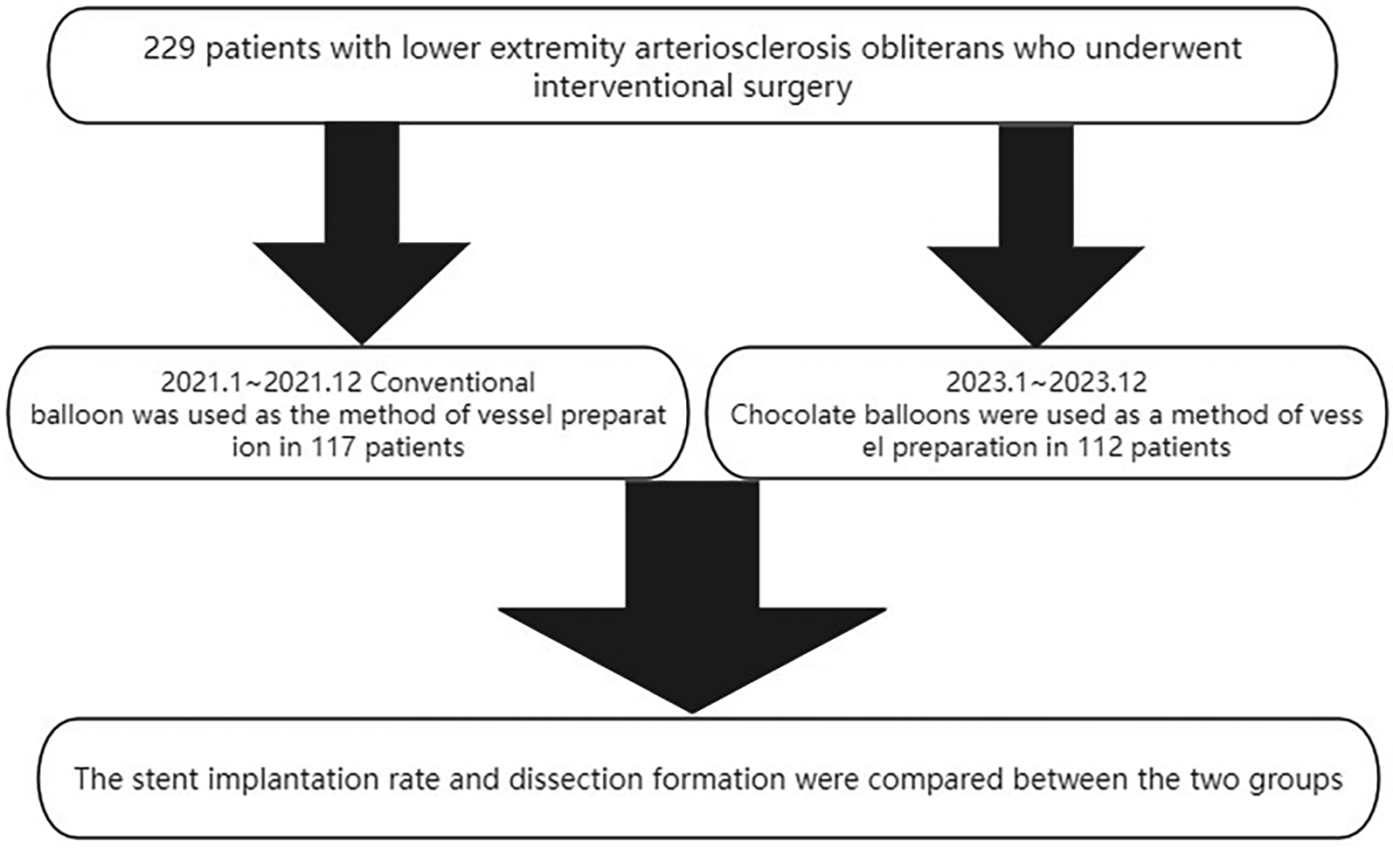
Study flowchart.

## 2 Data and methods

### 2.1 General information

A retrospective analysis was conducted on general data from our center, covering the period from January to December 2021. This analysis included 117 patients diagnosed with lower-limb arterial lesions. Additionally, data from January to December 2023 were collected from the same hospital, focusing on 112 patients with lower limb arterial lesions who underwent vascular preparation using chocolate balloons as a clinical intervention (Figures 1). (The study was approved by the Ethics Committee of the Center and was conducted in accordance with the 1964 Helsinki Declaration and its later amendments or comparable ethical standards).

### 2.2 Treatment method

**Figure 2:**
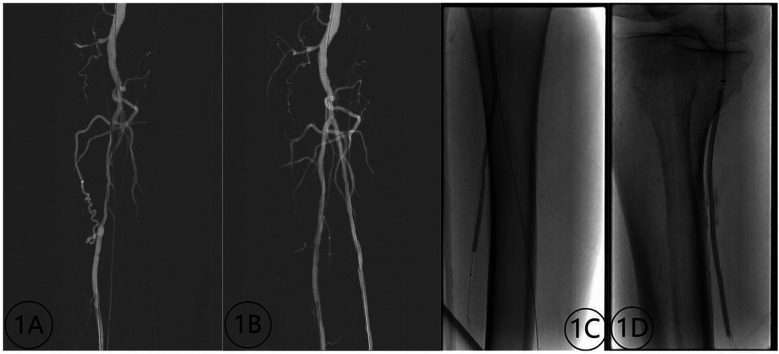
Contrast images before and after treatment of inferior knee artery occlusion (male, 72 years old) with a chocolate balloon. **(1A)** Preoperative image depicting stenosis and occlusion of the tibial and posterior peroneal arteries; **(1B)** Postoperative image displaying lumen patency without dissection; **(1C,D)** imaging demonstrating the chocolate balloon formation.

**Figure 3:**
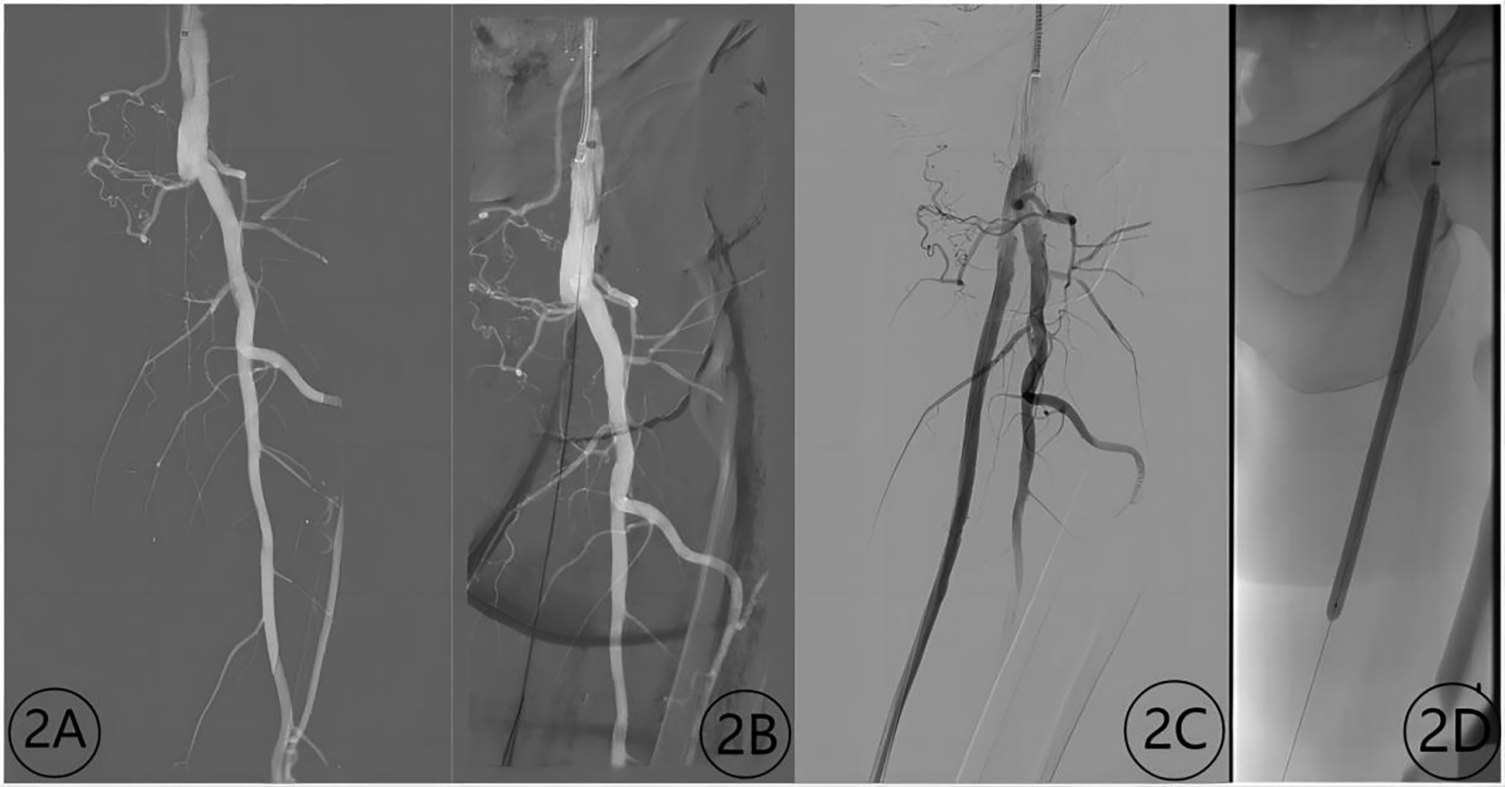
Contrast images before and after treatment with a chocolate balloon (male, 72 years old). **(2A)** Occlusion of the superficial femoral artery before treatment; **(2B)** guide wire passage through the occlusion segment; **(2C)** contrast image after treatment; **(2D)** image depicting the chocolate balloon formation.

**Figure 4:**
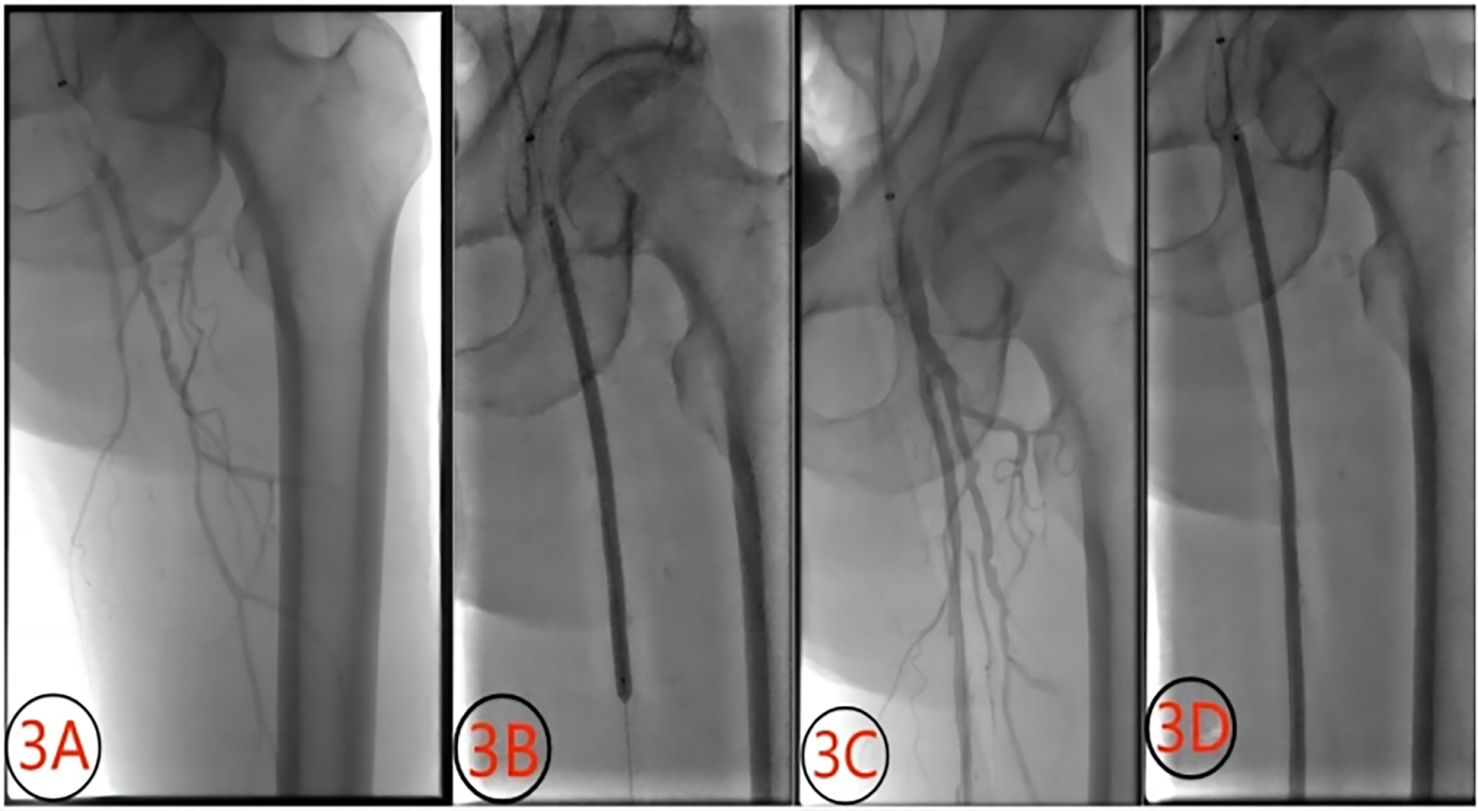
Contrast images before and after treatment with a chocolate balloon (male, 72 years old). **(3A)** superficial femoral artery occlusion before treatment; **(3B)** chocolate balloon dilation; **(3C)** post-dilation image showing non-flow-limiting dissection; **(3D)** image depicting dilation with DCB.

**Figure 5:**
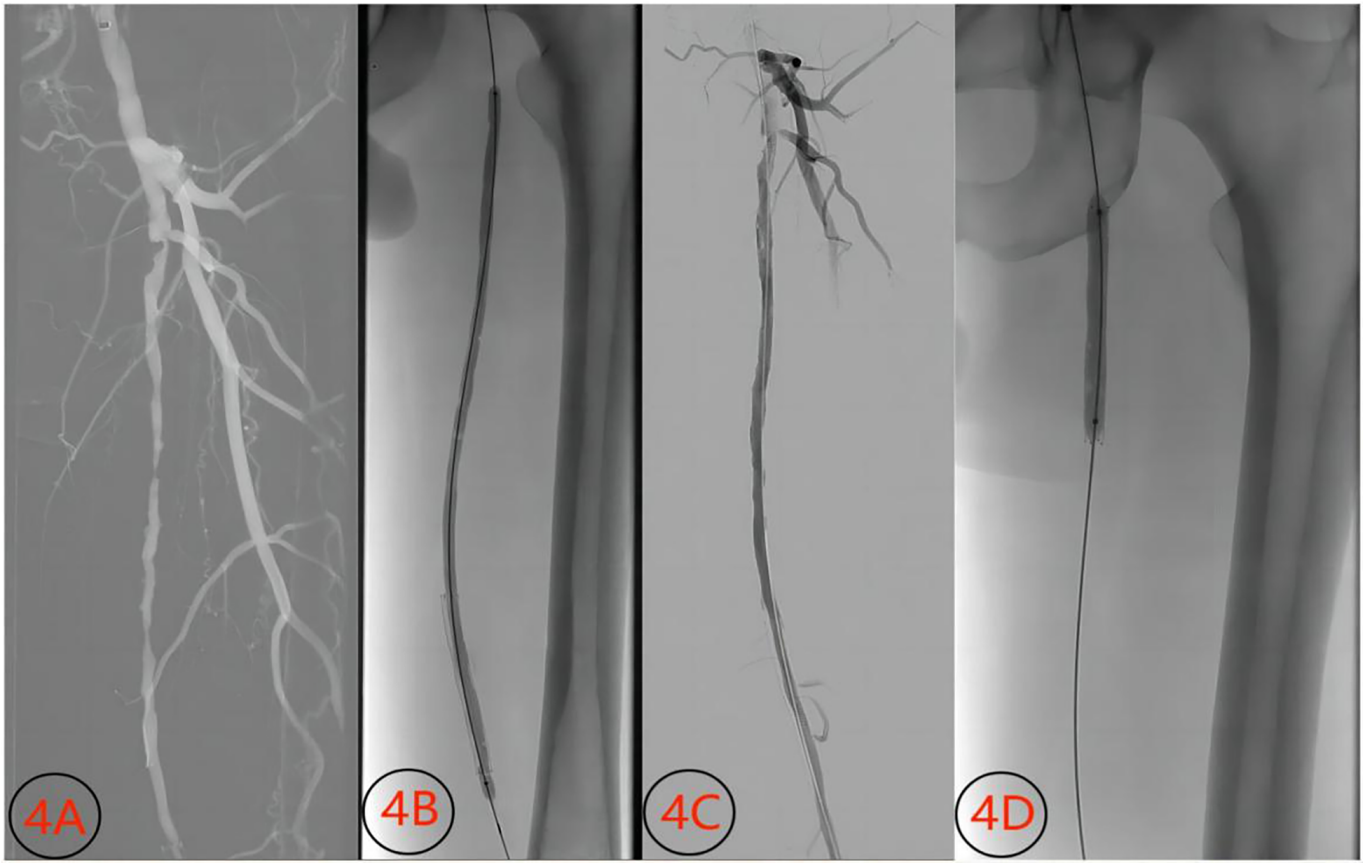
Images before and after treatment of superficial artery occlusion (male, 57 years old) with conventional balloon. **(4A)** Preoperative image showing short-segment occlusion and stenosis of the superficial femoral artery; **(4B)** conventional balloon dilatation; **(4C)** image showing dissection and significant stenosis after conventional balloon dilatation; **(4D)** Image showing balloon expansion and stent release.

**Figure 6:**
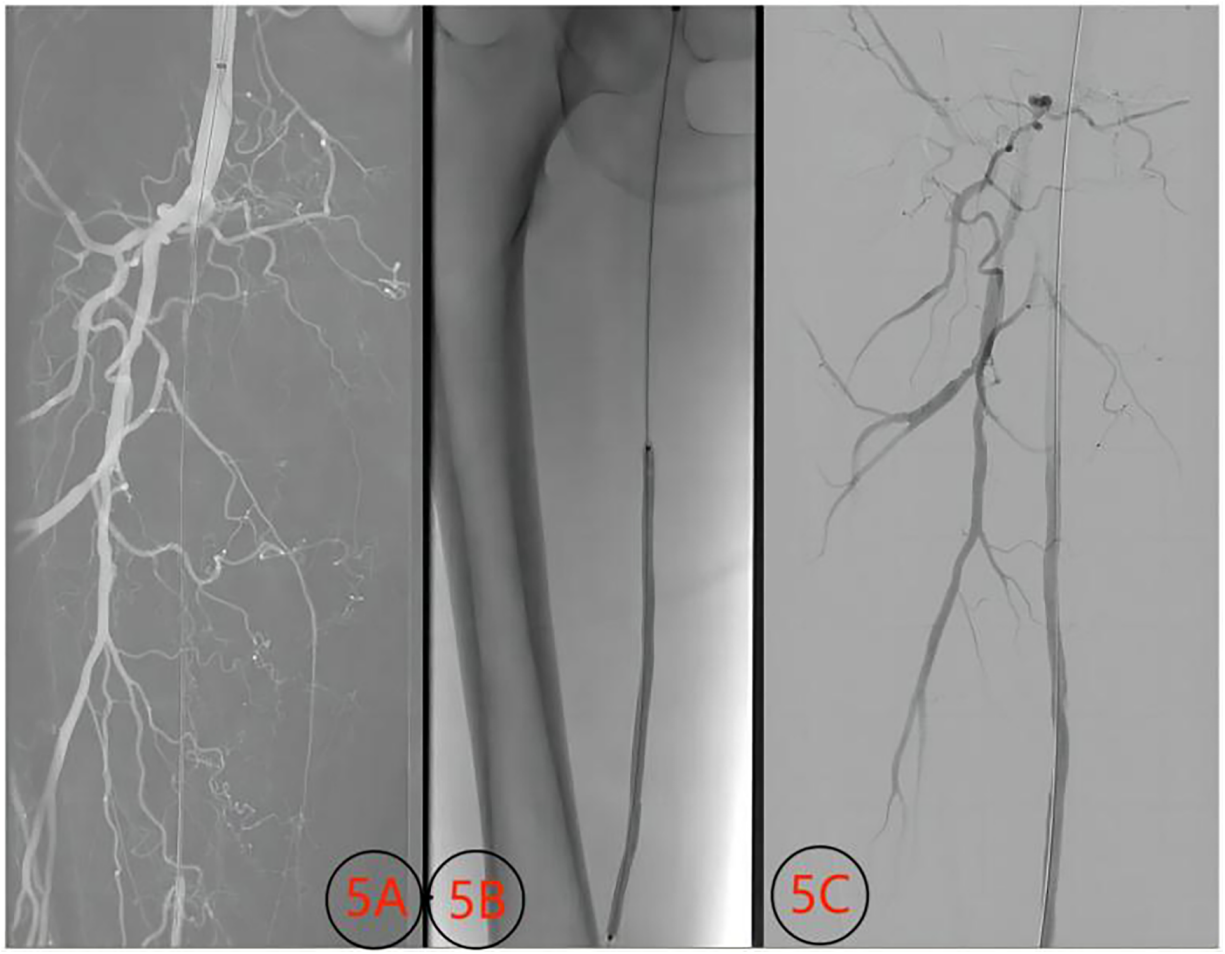
Images of superficial femoral artery thrombosis (female, 65 years old) with volume reduction technique and contrast images before and after conventional balloon treatment. **(5A)** Occlusion of the superficial femoral artery before treatment; **(5B)** conventional balloon dilation; **(5C)** angiography image after balloon dilation.

**Figure 7:**
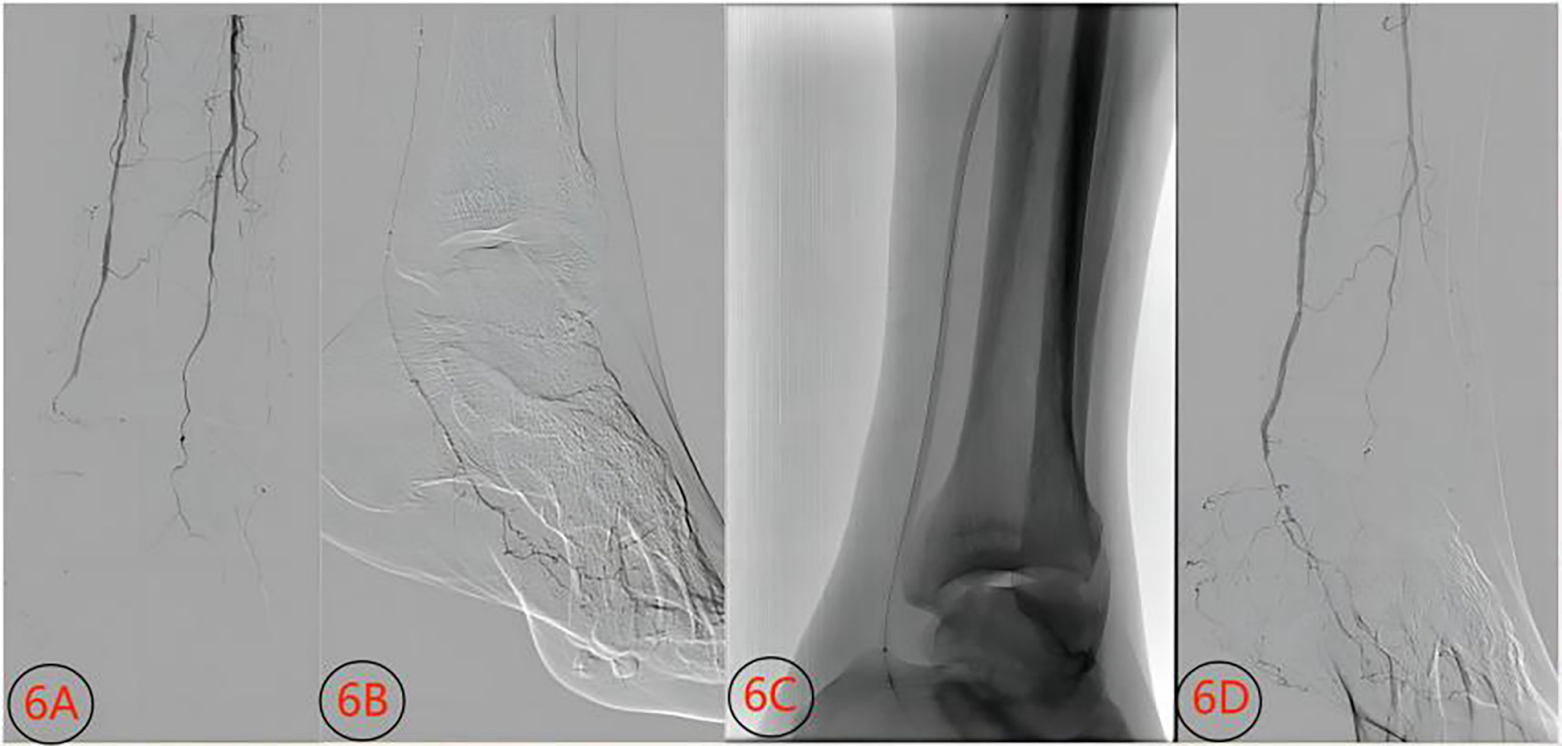
Contrast images before and after conventional balloon treatment for an inferior knee artery occlusion lesion (male, 74 years old). **(6A)** Stenosis and occlusion of the posterior tibial artery before treatment; **(6B)** guide wire passage through the distal posterior tibial artery; **(6C)** conventional balloon dilation; **(6D)** opening of the posterior stenosis segment after treatment.

The surgical pictures are shown in Figures 2–7. Patients were positioned supine for treatment, undergoing either an antegrade or retrograde puncture of the common femoral artery to facilitate the operation. Conventional local infiltration anesthesia was administered in the inguinal area. The Seldinger technique was utilized for femoral artery puncture, followed by the insertion of a 6F vascular sheath. Angiography was then performed to assess the lesion site, occlusion degree, lesion length, and distal outflow. Subsequent to systemic heparinization, a 4F single-bent catheter or support catheter of 0.035, 0.018, or 0.014 diameter was advanced through the lesion site. Subsequently, a chocolate balloon or a conventional balloon (manufacturers: Medtronic, Abbott, Zylox-Tonbridge, Acotec; length: 6–15 cm; pressure: 8–12 atm) was manipulated as the primary means of vessel preparation. Pre-dilatation with a small diameter balloon (2.5 mm) may be used for lesions that are difficult to pass. The appropriate balloon size was determined based on the vessel diameter at the lesion site. The chocolate balloon was inflated gradually to allow for a slow pressure rise, reaching 2 atmospheres (atm) within 30 to 60 s, followed by a slow, uniform increase to the standard pressure. Pressure maintenance at the standard level was sustained for 3 min after release using a pressure pump. Angiography was conducted following balloon expansion and dissection, with residual stenosis exceeding 50% or presenting as type D or worse, indicating the need for further treatment. In cases where this criterion was not met, DCB was chosen as the subsequent treatment modality.

### 2.3 Observing indicators

Dissection: Contrast images taken from multiple angles are used to assess any dissection formation. Dissections are classified according to the NHLBI classification of the National Heart, Lung, and Blood Institute in the United States. Severe dissections are categorized as type E and F dissections, while severe dissections encompass type D, E, and F (9).

The surgical success rate is calculated using the following formula: number of lesions with revascularization/total lesions = surgical success rate.

Stent implantation: Stent implantation refers to the direct placement of a stent following balloon dilatation.

### 2.4 Statistical analysis

Statistical analyses were performed using IBM® SPSS® Statistics software version 26.0. The measurement data were expressed as mean ± standard deviation (x ± s)and were compared using Student t test or Mann–Whitney *U-*test for non-normally distributed data. Numerical data are presented as numbers (percentages) and were compared using the chi-square test, Fisher exact, or Mann–Whitney *U*-test, with statistical significance set at *P* < 0.05.

## 3 Results

Baseline characteristics are shown in Table 1. Procedural characteristics are shown in Table 2. No significant differences were observed in baseline demographics, comorbidities, or lesion characteristics between the two groups (all *P* > 0.05), supporting the comparability of the cohorts. The success rate of surgery in both groups reached 100%. In the conventional balloon expansion group, the stent was directly implanted in 22 locations, while in the chocolate balloon expansion group, it was placed in 10 locations (*P* < 0.05). Following conventional balloon expansion, 21 interlayers were formed, comprising six non-flow-limiting and 15 severe interlayers, with 15 directly receiving scaffold implants. Conversely, after chocolate balloon expansion, 23 interlayers formed, including 17 non-flow-limiting and six severe interlayers, with six directly receiving stent implants. Baseline data are shown in Table 1, revealing no statistically significant differences in patient demographics or lesion characteristics. Surgical outcomes and patient results are shown in Table 2. In the Chocolate balloon group vs. the conventional balloon group, the rates were as follows: overall dissection (20.5% vs. 17.5%, *P* = 0.619 > 0.05), non-flow-limiting dissection (14.7% vs. 4.8%, *P* < 0.05), flow-limiting dissection (5.8% vs. 12.7%, *P* < 0.05), and stent implantation (9.0% vs. 18.3%, *P* < 0.05).

**Table 1:** Patient baseline data.

Patient data	Chocolate	Conventional	*P*
Age (x ± s, years)	71.93 ± 8.714	71.09 ± 8.606	0.462
Male: Female	68:44	66:51	0.509
Body mass index (x ± s, kg/m^2^)	23.448 ± 3.129	23.096 ± 3.232	0.404
Risk factor			
Hypertension	88 (78.571)	84 (71.795)	0.236
High blood lipids	36 (32.143)	34 (29.060)	0.613
Type 2 Diabetes Mellitus	73 (65.179)	77 (65.812)	0.920
CKD	7 (6.25)	9 (7.692)	0.669
Smoking	88 (78.571)	84 (71.795)	0.138
Rutherford			
Level 1	0 (0)	1 (0.855)	0.329
Level 2	3 (2.679)	4 (3.419)	0.745
Level 3	28 (25)	30 (25.641)	0.911
Level 4	30 (26.786)	21 (17.949)	0.108
Level 5	49 (43.75)	46 (39.316)	0.496
Level 6	10 (8.929)	15 (12.821)	0.345
Anticoagulants	7 (6.25)	9 (7.692)	0.669
Antiplatelet Agents	51 (45.536)	54 (46.154)	0.925
Lesion length (x ± s, cm)	19.734 ± 9.843	21.667 ± 10.891	0.161
Type of lesion			
CTO lesions	85 (75.893)	80 (68.376)	0.874
Stenotic lesions	6 (5.357)	12 (10.256)	0.168
Thrombotic lesions	16 (14.286)	25 (21.368)	0.162
PACSS			
Level 0	4 (3.571)	4 (3.419)	0.970
Level 1	41 (36.607)	45 (38.462)	0.772
Level 2	25 (22.321)	26 (22.222)	0.986
Level 3	25 (22.321)	22 (18.803)	0.510
Level 4	17(15.179)	20(17.094)	0.694

**Table 2:** Patient surgical outcomes.

Surgical data	Chocolate	Conventional	*P*
Application of lesion location			
Above the knee	85 (76.2)	89 (72.2)	0.975
Below the knee	24 (19.7)	23 (18.2)	0.740
Mix	3 (2.5)	5 (7.9)	0.511
Chocolate or Conventional	112	117	
Dissection	23 (20.5)	21 (17.5)	0.619
Severe dissection	6 (5.8)	15 (12.7)	0.003
Final device			
Drug-coated balloon	102 (91.0)	95 (81.7)	0.031
Placement stent	10 (9.0)	22 (18.3)	0.031

## 4 Discussion

Currently, balloon dilatation and stent implantation stand as primary treatments for lower limb arterial occlusive disease (10). While stent implantation yields short-term vascular patency, mounting concerns arise regarding its medium- and long-term efficacy (11). Acting as an external foreign body, stents inflict damage on vascular endothelial cells, provoke inflammation, and induce platelet aggregation, leading to intrastent restenosis, displacement, and rupture, and thus constraining their application (12–16). Meanwhile, balloon dilation of blood vessels subjects them to uneven dilation and secondary radial, torsional, and longitudinal shear stress, resulting in varying degrees of vascular damage that impede long-term efficacy (17). Effective vascular preparation aims to minimize wall damage, reduce severe dissections, counter significant elastic retraction, and enhance drug penetration from DCB into the vascular wall. Numerous studies attest to the inhibitory effects of DCB-contained drugs on vascular smooth muscle cell hyperplasia. However, conventional balloons, often used for vascular preparation, are associated with a high incidence of dissection and subsequent stent implantation (18–20). Hence, successful drug delivery via DCB hinges on adequate vascular preparation. This involves pretreating lesions to ensure sufficient predilation, minimal residual stenosis, and optimal dissection flow, thereby furnishing an optimal vascular inner wall surface for drug delivery and ensuring effective drug release.

In interventional surgery, insufficient vascular preparation often results in dissection formations, a primary factor prompting stent implantation. Vascular dissection primarily stems from damage or even tearing induced by uneven stress on the vascular intima. A study by Moriwaki et al. (21) examining the impact of balloon pressure on blood vessel walls revealed disparate pressure effects on normal elastic vs. sclerotic blood vessels. Varying pressure differentials can readily lead to endometrial injury due to pneumatic pressure. During balloon dilation, a portion of the balloon typically expands and forms first, followed by subsequent expansion. Additional pressure is often required to fully inflate the balloon, resulting in further, uncontrollable damage to the preformed blood vessels. Moreover, a stepped pressure differential commonly occurs at the junction between preformed and post-formed blood vessels, leading to vascular wall displacement.

With the uncontrolled escalation of balloon pressure, the vascular intima at the junction of blood vessel walls can become misaligned and torn, increasing the likelihood of forming severe dissections. The unique nickel-titanium wire of the chocolate balloon restricts the structure of the balloon, enabling controlled and uniform expansion. This promotes plaque improvement while aiming to minimize stress changes and vascular wall damage. The restraining filament divides the expanding balloon, creating grooves on its surface. Consequently, the restraining structure forms a pattern of channels, or “pillows”, on the balloon as it reaches its maximum expansion. The “pillows” touch the blood vessel wall, distributing pressure, reducing dissection, and facilitating expansion. Through this mechanism, along with secondary expansion, prolonged expansion duration, and adjustments to the contact position of the “pillows”, shear stress can be minimized, ensuring uniform, consistent, and thorough expansion to maximize benefits while effectively reducing intercalation and the need for stent implantation. Unlike other vascular preparation methods such as notch balloons, conventional balloons, cutting balloons, shock wave balloons, and plaque rotary techniques, the chocolate balloon does not necessitate the absolute true cavity passage of the guidewire. It achieves effective dilation by passing beneath the intima, offering superior dilation effects.

In the observational studies conducted by various researchers, the benefits of using chocolate balloons become evident. For instance, in the chocolate BAR study conducted by Muatapha et al. (8), 262 patients with superior knee arteriosclerosis obliterative disease underwent treatment with chocolate balloons. A remarkable 85.1% of these patients reached the primary endpoint, demonstrating surgical success. The need for remedial stent implantation was a mere 1.6%, highlighting the efficacy of short-term operations employing chocolate balloons. Similarly, a single-center retrospective study by Shirai et al. (22) examined 111 patients with femoral popliteal artery lesions. A comparison between two vascular preparation strategies, chocolate balloon combined with DCB vs. conventional balloon combined with DCB, revealed significant differences. The chocolate balloon group exhibited a severe dissection rate of 4.2%, significantly lower than the 25% in the conventional balloon group. Additionally, the diameter stenosis rates after expansion were 18 ± 15% and 20 ± 17% for the chocolate and conventional balloon groups, respectively. The stent implantation rates were 2.1% and 15.9%, respectively, with the main patency rates at six months being 89.1% and 85.2%, respectively. Another study by Sirignano et al. (23) reported a remedial stent implantation rate of 9.5%. Moreover, a meta-analysis conducted by Giannopoulos et al. (24) involving 3,029 patients with various lesion types averaging 26.9 cm in length revealed significant advantages of special balloon and vessel volume reduction combined with DCB over conventional balloon angioplasty (plain old balloon angioplasty; POBA) in increasing patency rate and reducing stent implantation and restenosis. These findings collectively underscore the efficacy of chocolate balloons across diverse lesion types compared to POBA. They facilitate optimal vascular preparation conditions for DCB use, while maintaining favorable dilatation effects.

This study encompassed 117 patients with lower extremity arterial disease treated with conventional balloons between January 2021 and December 2021, along with 112 patients treated with chocolate balloon catheters between January 2023 and December 2023 in our center. Both groups presented with CTO lesions, stenosis lesions, and thrombotic lesions. Notably, in the chocolate balloon group, 84.8% of the patients exhibited CTO lesions, with 37.4% having severe calcified lesions (peripheral arterial calcium scoring system;PACSS III and IV). Conversely, in the conventional balloon group, 68.4% exhibited CTO lesions, with 35.9% presenting severe calcified lesions (PACSS III and IV). The rates of severe entrapment formation were 5.8% and and 12.7% in the chocolate and conventional groups, respectively (*P* < 0.05), and the rates of stent implantation were 9.0% and 18.3%, respectively (*P* < 0.05). In this study, the stent implantation rate in both groups surpassed that observed in other studies. This discrepancy can be attributed to the significantly lower prevalence of CTO lesions (31.3%) and severely calcified lesions (10.5%) reported in the Chocolate BAR study compared to our center's patient demographics. Furthermore, studies have indicated that severe calcification independently increases the risk of postoperative restenosis (25). For instance, in Shirai et al.'s study, severe calcified lesions comprised 51.3% of cases, with CTO lesions accounting for 29.7%.

In Sirignano et al.'s study (23), CTO lesions comprised 65.5% of cases, yet fall short of the proportion observed in our center. Within our center, the application of chocolate balloons is guided by accumulated experience, emphasizing meticulous handling during initial balloon expansion to minimize barotrauma. This entails a gradual pressure increase, reaching 2 atm within 30 to 60 s, and achieving standard pressure at a slow, uniform pace. Multiple expansions may be necessary for optimal results, allowing for adjustments in decompression tank positioning to ensure uniform expansion, particularly in cases of severe calcification. Gradually escalating expansion pressure, potentially nearing the maximum burst pressure, proves essential for combating elastic retraction, especially with severe calcification. The severity of calcification dictates the duration of full balloon filling, mitigating barotrauma. However, limitations of the chocolate balloon have surfaced in our center's practice. As a non-vascular debulking technique, it struggles to address elastic retraction in severely calcified lesions, particularly those beneath the intima. Furthermore, the 0.014 guidewire system, standard in specifications below 5 mm, poses challenges in navigating CTO lesions without predilation. This study, conducted within a single center with a limited sample size, solely evaluates the immediate postoperative outcomes of multi-segmental and multi-morphological lesions, focusing on dissection incidence and stent implantation rates during the procedure. Further study is still needed to observe the long-term vascular patency rates associated with chocolate balloons.

In summary, the chocolate balloon emerges as a superior vessel preparation option compared to the conventional balloon. Moreover, it mitigates the demanding nature of other techniques for navigating the true lumen. In the real world, the chocolate balloon proves adept at providing optimal vessel preparation across various types of lower extremity arterial diseases. By establishing favorable vascular conditions for subsequent endovascular treatments, it effectively diminishes the incidence of severe dissection and necessitates fewer instances of stent implantation. These attributes underscore its significant clinical utility.

## Data Availability

The raw data supporting the conclusions of this article will be made available by the authors, without undue reservation.
